# Neuropsychiatric Presentation of Non-paraneoplastic Voltage-Gated Calcium Channel Autoimmune Encephalitis: A Case Report

**DOI:** 10.7759/cureus.102960

**Published:** 2026-02-04

**Authors:** Shreya Philip, Sandeep Kaur, Rajeshwar Sahonta, Jeyaraj D Pandian

**Affiliations:** 1 Department of Neurology, Christian Medical College and Hospital, Ludhiana, IND

**Keywords:** autoimmune encephalitis and atypical presentations, intravenous immunoglobulins (ivig), neuro-psychiatric, passive immunotherapy, voltage-gated calcium channel antibody

## Abstract

Autoimmune encephalitis is an immune-mediated inflammatory disorder of the brain with diverse neurological and psychiatric manifestations. Voltage-gated calcium channel (VGCC) antibody-associated autoimmune encephalitis is rare and has been predominantly described in paraneoplastic settings. Emerging reports suggest a broader range of central nervous system involvement, but presentations dominated by psychiatric symptoms remain uncommon and diagnostically challenging.

A 39-year-old man with no prior medical illness presented with subacute progressive cognitive decline, behavioral changes, and religious-themed complex auditory hallucinations over three to four months. Initial evaluation, including magnetic resonance imaging, electroencephalography, and standard autoimmune encephalitis antibody testing, was unrevealing. Extended cerebrospinal fluid and serum autoimmune testing were done, which demonstrated elevated VGCC antibody levels. Comprehensive malignancy screening, including whole-body positron emission tomography, showed no evidence of an underlying neoplasm. The patient showed partial improvement following intravenous methylprednisolone and intravenous immunoglobulin (IVIG). Symptom recurrence necessitated repeat IVIG and escalation to rituximab therapy, resulting in sustained clinical improvement.

This case broadens the recognized clinical spectrum of VGCC antibody-associated autoimmune encephalitis by highlighting a presentation dominated by psychiatric symptoms in the absence of malignancy or classical neurological features. Recognition of such atypical presentations is essential, as early diagnosis and appropriate immunotherapy can lead to favorable outcomes.

## Introduction

Autoimmune encephalitis comprises a group of disorders where the immune system targets neuronal surface, synaptic, or intracellular antigens, resulting in brain inflammation. Patients typically present with subacute neuropsychiatric symptoms, including cognitive dysfunction, behavioral changes, seizures, and psychiatric features such as hallucinations or delusions, which may precede or dominate the clinical presentation [1].

Autoimmune encephalitis is classified based on the location of the antigen. One group involves antibodies against neuronal surface antigens (e.g., N-methyl-D-aspartate (NMDA) and α-amino-3-hydroxy-5-methyl-4-isoxazolepropionic acid (AMPA) receptors), while another targets intracellular paraneoplastic markers (e.g., anti-neuronal nuclear antigen type 1 (Hu/ANNA-1), Purkinje cell cytoplasmic antibody type 1 (Yo), anti-neuronal nuclear antigen type 2 (Ri/ANNA-2), Collapsin Response Mediator Protein 5 (CRMP5), paraneoplastic antigen Ma2 (PNMA2/Ma2), Amphiphysin, and Delta/Notch-like epidermal growth factor-related receptor (Tr) [2]. Other categories include antibodies against synaptic proteins or undefined targets associated with inflammatory syndromes [3].

Recent advances have identified multiple antibodies-such as those against the NMDA receptor, voltage-gated potassium channel (VGKC) complex (Leucine-Rich Glioma-Inactivated protein 1 (LGI1), Contactin-Associated Protein-Like 2 (CASPR2)), gamma-aminobutyric acid type A receptor (GABA-A), gamma-aminobutyric acid type B receptor (GABA-B), AMPA receptor, immunoglobulin-like cell adhesion molecule LON5 (IgLON5), glutamic acid decarboxylase 65-kDa isoform (GAD65), Ma2, Hu, voltage-gated calcium channel (VGCC)-and their related clinical syndromes, contributing to the expanding field of autoimmune neurology [4].

Although VGCC antibodies are well established in paraneoplastic neurological syndromes, their role in primary autoimmune encephalitis and isolated neuropsychiatric presentations remains incompletely understood. VGCCs, widely expressed in the central nervous system, are essential for neuronal signaling and synaptic transmission. Antibodies against P/Q- and N-type VGCCs are classically associated with Lambert-Eaton myasthenic syndrome, often linked to small-cell lung cancer, but have also been reported in paraneoplastic and non-paraneoplastic cerebellar degeneration. [5]

Central nervous system involvement is rare and heterogeneous, with manifestations that may include cognitive impairment, limbic and extralimbic encephalopathy, cerebellar ataxia, and peripheral nervous system features. Neuroimaging, when abnormal, may show limbic or extralimbic cortical changes, such as gyriform enhancement, cortical laminar necrosis, or isolated hippocampal involvement [6-8].

Predominant or isolated neuropsychiatric presentations in VGCC-antibody-associated autoimmune encephalitis are exceptionally uncommon and may lead to misdiagnosis as a primary psychiatric disorder, delaying appropriate immunotherapy. We describe a case of VGCC-antibody-associated autoimmune encephalitis presenting with subacute confusion, visual hallucinations, paranoid delusions, and catatonia-like features, underscoring the expanding clinical spectrum of this rare autoimmune condition.

## Case presentation

A 39-year-old man with no prior medical comorbidities presented with subacute, progressive cognitive decline, behavioural changes, hallucinations, and headache evolving over three to four months. During this period, he became increasingly withdrawn, with a marked reduction in spontaneous speech and social interaction. He was noted to remain in fixed, awkward postures for prolonged durations and did not change position despite encouragement. He also reported complex auditory hallucinations with a religious theme.

The patient was evaluated at two outside centers, where cerebrospinal fluid (CSF) autoimmune antibody testing using a cell-based immunofluorescence assay on undiluted CSF (including NMDA, AMPA, glutamate receptor 1 (GluR1), glutamate receptor 2 (GluR2), GABA-B, LGI1, and CASPR2 antibodies) was negative. He received a five-day course of intravenous methylprednisolone (1 g/day), following which partial clinical improvement was observed. However, symptoms recurred after a brief interval, leading to his presentation to our institution for further evaluation and management.

On examination, the patient was alert and oriented. Higher mental functions were assessed and were normal. Neurological examination revealed grade I spasticity with brisk deep tendon reflexes (3+), while cerebellar function was preserved. Based on the clinical presentation, differential diagnoses considered included autoimmune encephalitis, primary psychotic disorder, catatonia, paraneoplastic neurological syndromes, and central nervous system vasculitis. A trial of risperidone was initiated for psychotic symptoms, but no clinical improvement was observed.

Baseline laboratory investigations, including complete blood counts, liver and renal function tests, and inflammatory markers (erythrocyte sedimentation rate: 2 mm/hr; C-reactive protein: 0.2 mg/dL), were within normal limits. Serological testing for human immunodeficiency virus (HIV), hepatitis B, and hepatitis C was negative. The vasculitis workup demonstrated weakly positive antinuclear antibodies and a mildly elevated cytoplasmic antineutrophil cytoplasmic antibody (c-ANCA) level of 17.7. Tumor markers, including alpha-fetoprotein, cancer antigens CA 15-3, CA 19-9, and carcinoembryonic antigen (CEA), were within normal limits. Paraneoplastic antibody testing revealed borderline positivity for Sry-like high-mobility group box 1 (SOX-1) antibodies.

Magnetic resonance imaging (MRI) of the brain

**Figure 1:**
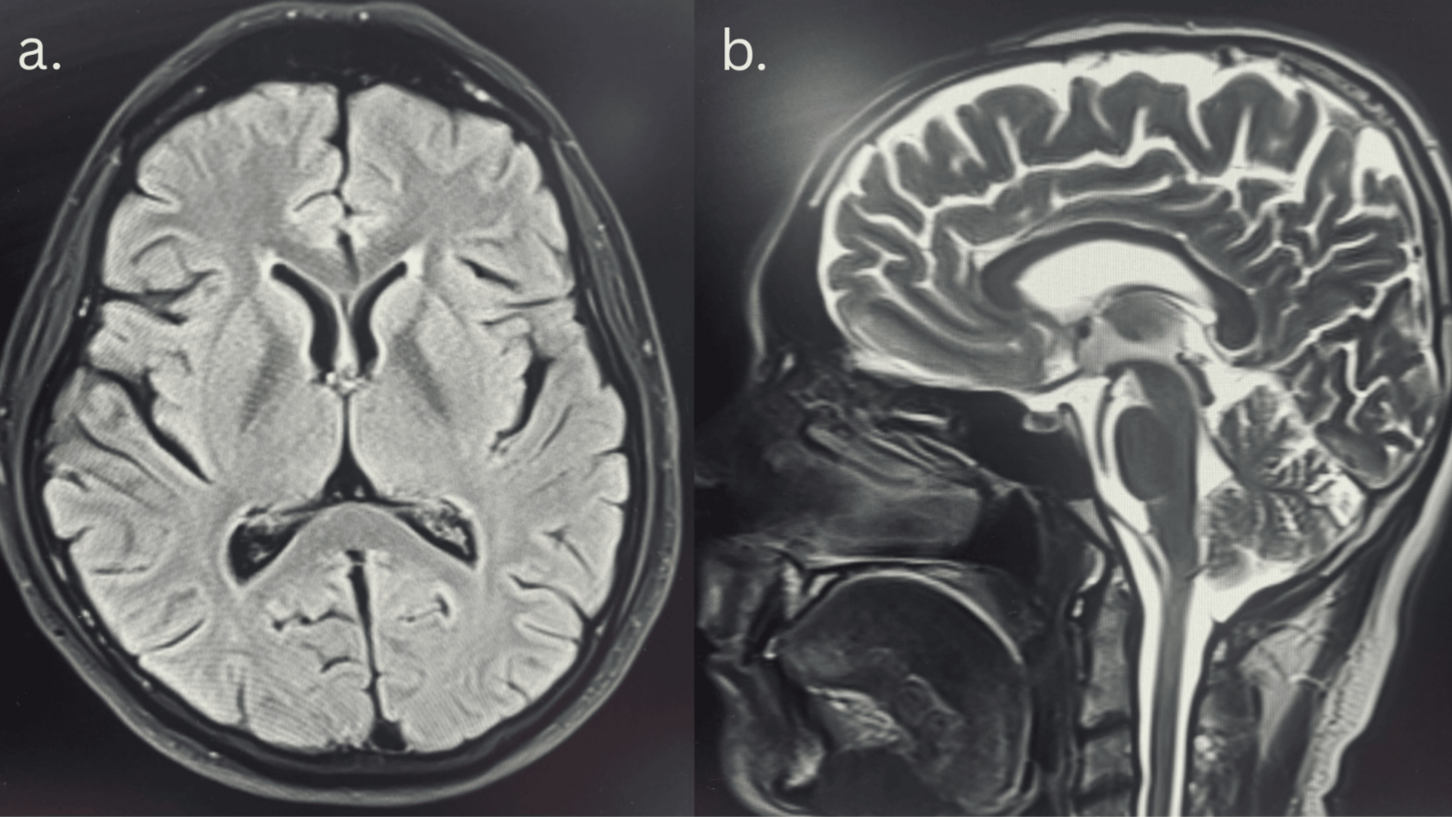
(a) MRI axial FLAIR and (b) sagittal T2-weighted (right) images of this patient with VGCC autoimmune encephalitis, showing no abnormal signal or enhancement. MRI, magnetic resonance imaging; FLAIR, fluid-attenuated inversion recovery; VGCC, voltage-gated calcium channel

Magnetic resonance (MR) angiography and venography did not reveal any structural or vascular abnormalities (Figure 1).

An electroencephalogram (EEG) was performed using the International 10-20 electrode placement system with 16 channels, employing both referential and bipolar montages. Recording parameters included a sensitivity of 7 µV/mm, a high-pass filter of 1.6 Hz, a low-pass filter of 70 Hz, and standard notch filtering. The patient remained awake throughout the recording, and activation procedures, including hyperventilation and intermittent photic stimulation, were performed without inducing epileptiform activity.

Background activity consisted of a bilaterally symmetrical and synchronous alpha rhythm at 8-10 Hz with amplitudes of 20-30 µV, maximal over both occipital regions, and appropriately attenuated with eye opening. There was no evidence of spikes, sharp waves, or epileptiform discharges. No voltage asymmetry, wave asymmetry, phase reversal, or lateralizing epileptiform focus was observed. Additionally, there were no EEG features suggestive of nonconvulsive status epilepticus or focal slowing.

**Figure 2:**
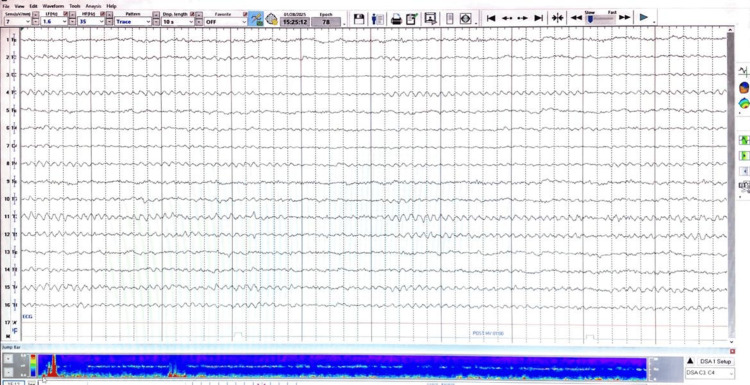
EEG tracing of this patient with VGCC autoimmune encephalitis, showing normal background activity without epileptiform discharges. EEG, electroencephalogram; VGCC, voltage-gated calcium channel

The EEG was normal during wakefulness, with no evidence of epileptiform abnormalities (Figure 2).

Computed tomography (CT) angiography of the brain and neck, along with CT perfusion imaging, did not demonstrate any vascular or perfusion abnormalities. Transthoracic echocardiography was unremarkable. CSF analysis revealed a protein concentration of 43.25 mg/dL and a glucose level of 79.4 mg/dL, with an adenosine deaminase value of 6 U/L. The CSF cell count showed no red or white blood cells. CSF cultures showed no growth, and there was no evidence of cryptococcal or acid-fast bacilli infection. On India ink, no capsulated budding yeast-like cells were seen. CSF electrophoresis did not demonstrate oligoclonal bands. Both CSF and serum samples were sent to the Amrita Institute of Medical Sciences and Research Centre for comprehensive autoimmune antibody evaluation. The extended panel included 23 antibodies, encompassing NMDAR, CASPR2, IgLON5, GABA-A, GABA-B, LGI1, AMPA, and VGCC antibodies. The results were positive for VGCC antibodies at a level of 270 pg/mL (reference range <149 pg/mL). Antibody testing was performed using an indirect immunofluorescence assay on undiluted CSF and serum diluted at 1:10. The results of the CSF analysis are depicted in Table 1. 

**Table 1:** Laboratory evaluation demonstrating CSF analysis findings. CSF, cerebrospinal fluid; RBC, red blood cell; WBC, white blood cell

Test name	Value	Biological reference range
Protein (mg/dL)	43.25	15-40 mg/dL
Glucose (mg/dL)	79.4	50-80 mg/dL
Adenosine deaminase (U/L)	6	Less than 10 U/L
Oligoclonal bands	Not detected	Negative bands
CSF cell count	RBC - Nil; WBC - Nil	RBC - 0 RBCs/µL; WBC - 0-5 WBCs/µL
Cultures	No growth, no acid-fast bacilli seen	-
India ink	No capsulated budding yeast-like cells seen	-
Cryptococcal antigen	Negative	-

The results of the autoimmune antibody testing are elaborated in Table 2. 

**Table 2:** Autoimmune workup including serum and CSF antibody profile of the patient. CSF, cerebrospinal fluid; MA2/TA, PNMA2 (paraneoplastic neuronal antigen 2) protein/tumor antigen; GABA A receptor, gamma-aminobutyric acid type A receptor; GFAP, glial fibrillary acidic protein; DPPX 6, dipeptidyl-peptidase-like protein 6; IgLON5, immunoglobulin-like cell adhesion molecule 5; NMDA, N-methyl-D-aspartate; AMPA, α-amino-3-hydroxy-5-methyl-4-isoxazolepropionic acid; AMPA 1 receptor, targets the GluA1 subunit of the AMPA receptor; AMPA 2 receptor, targets the GluA2 subunit of the AMPA receptor; GABA B receptor, gamma-aminobutyric acid type B receptor; Zic 4, zinc finger protein of the cerebellum 4

Test	Value	Reference range
Antineuronal nuclear antibody-1 (ANNA-1/anti-Hu) in serum	Negative	Negative
Antineuronal nuclear antibody-2 (ANNA-2/anti-Ri) in serum	Negative	Negative
Antineuronal nuclear antibody-3 (ANNA-3) in serum	Negative	Negative
Antiglial nuclear antibody-1 (AGNA-1) in serum	Negative	Negative
Purkinje cell cytoplasmic antibody-2 (PCA-2-Tr) in serum	Negative	Negative
Purkinje cell cytoplasmic antibody (PCA-Tr) in serum	Negative	Negative
Amphiphysin antibody in serum	Negative	Negative
Collapsin response mediator protein-5 (CRMP-5) in serum	Negative	Negative
MA2/TA antibody in serum	Negative	Negative
GABA A receptor antibody in serum	Negative	Negative
GFAP antibody in CSF	Negative	Negative
DPPX 6 antibody in serum	Negative	Negative
Glycine receptor antibody in serum	Negative	Negative
IgLON5 antibody in serum	Negative	Negative
NMDA receptor antibody in CSF	Negative	Negative
Contactin-associated protein 2 (CASPR2) antibody in serum	Negative	Negative
Leucine-rich glioma inactivated protein 1 (LGl1-VGKC-associated) antibody in serum	Negative	Negative
AMPA 1 receptor antibody in CSF	Negative	Negative
AMPA 2 receptor antibody in CSF	Negative	Negative
GABA B receptor antibody in CSF	Negative	Negative
Zic 4 antibody in serum	Negative	Negative
Voltage-gated calcium channel (VGCC ) antibody quantitation in serum	270 pg/mL	>149 pg/mL positive
Unclassified neuronal antibody	Negative	Negative

A whole-body positron emission tomography (PET) scan did not reveal any abnormal hypermetabolic lesions suggestive of an underlying malignancy.

The patient was treated with intravenous immunoglobulin (IVIG) at a total dose of 2 g/kg administered over five days, which was well tolerated and resulted in partial clinical improvement. He was subsequently discharged on tapering oral corticosteroids. However, one month later, symptom recurrence prompted readmission, and a second course of IVIG was administered over five days, along with initiation of rituximab (500 mg). The patient demonstrated gradual clinical improvement and was discharged on tapering oral steroids.

At the six-month follow-up, repeat PET-CT imaging and malignancy screening remained negative. The patient continues to be clinically stable on maintenance rituximab and low-dose oral corticosteroids.

**Figure 3:**
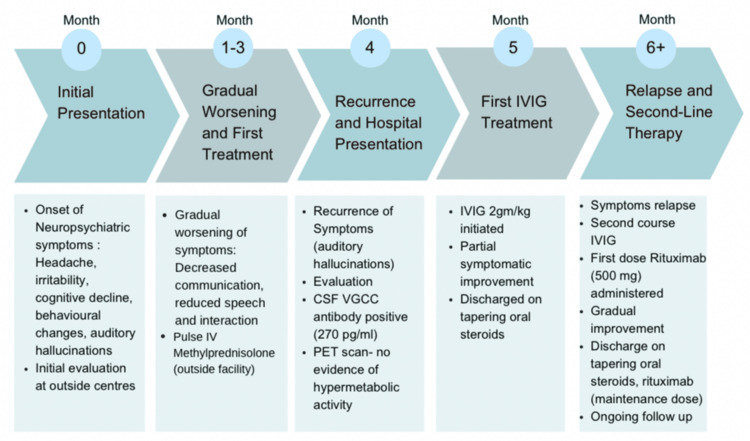
Clinical timeline of symptoms, interventions, and outcomes in this case of VGCC-antibody-mediated autoimmune encephalitis. Image credit: All authors VGCC, voltage-gated calcium channel; IV, intravenous; CSF, cerebrospinal fluid; PET, positron emission tomography; IVIG, intravenous immunoglobulin

A summary of symptom progression and therapeutic interventions is depicted in Figure 3.

## Discussion

This case illustrates an uncommon presentation of VGCC antibody-associated autoimmune encephalitis manifesting predominantly with psychiatric symptoms and subacute cognitive decline. The absence of seizures, focal neurological deficits, or supportive findings on magnetic resonance imaging and electroencephalography made the diagnosis challenging and initially raised the possibility of a primary psychiatric disorder.

Autoimmune encephalitis is now being more frequently recognized as a cause of neuropsychiatric presentations and its detection is increasing with new autoantibody biomarker discovery. However, the overall diagnosis remains rare [9, 10]. Autoimmune encephalitis should be considered in patients with subacute neuropsychiatric symptoms, particularly when the clinical course is progressive, atypical, or treatment-refractory psychiatric or cognitive symptoms, particularly when the presentation does not fit a primary psychiatric disorder.

VGCC antibodies are rare in autoimmune encephalitis and are less well characterized than antibodies such as NMDAR, LGI1, or CASPR2. They have a fundamental role in maintaining neuronal excitability and synaptic function. Although classically described in paraneoplastic contexts, particularly Lambert-Eaton myasthenic syndrome, similar antibodies have also been reported in non-paraneoplastic conditions such as cerebellar degeneration and ataxia [11-14].

Predominant psychiatric manifestations, including hallucinations and delusions, are exceptionally uncommon in VGCC-antibody-associated autoimmune encephalitis. In this case, the combination of a psychiatric-dominant clinical phenotype, lack of meaningful response to antipsychotic therapy, and partial improvement with immunotherapy favored an immune-mediated process. The diagnostic suspicion was further strengthened by the identification of elevated VGCC antibody titers on extended autoimmune evaluation, performed after initial standard antibody panels were unrevealing. Notably, normal cerebrospinal fluid parameters, neuroimaging, and electroencephalographic findings do not exclude autoimmune encephalitis and have been described in a subset of affected patients. Comprehensive malignancy screening remained negative, supporting a non-paraneoplastic form of VGCC-antibody-associated autoimmune encephalitis.

From a therapeutic standpoint, the patient exhibited transient improvement following corticosteroids and intravenous immunoglobulin, with subsequent relapse necessitating escalation to B-cell-directed therapy with rituximab. Sustained clinical stabilization following immunomodulatory treatment supports the pathogenic relevance of VGCC antibodies in this case and underscores the importance of timely and adequate immunosuppression in relapsing or treatment-refractory disease. IVIGs are medicines that help regulate the immune system and are used to treat many autoimmune and infectious diseases. They mainly contain immunoglobulin G (IgG), with a small amount of immunoglobulin A (IgA). The way IVIG works depends on the dose given. At low doses, especially in patients with immune deficiency, IVIG acts as a replacement for missing antibodies. At high doses, IVIG actively modifies the immune response and also reduces inflammation [15].

The diagnosis of autoimmune encephalitis is particularly challenging when psychiatric symptoms dominate the clinical presentation, often leading to diagnostic delays and increased morbidity. While immunotherapy remains the cornerstone of treatment, its indiscriminate use carries the risk of significant adverse effects [16]. Therefore, careful patient selection, judicious choice of agents, and appropriate treatment duration are essential to avoid both under- and overtreatment [17].

The strengths of this report include the rarity of the presentation, cerebrospinal fluid-based antibody confirmation, adherence to guideline-supported immunotherapy, and longitudinal follow-up. Limitations include the detection of VGCC antibodies in serum only, with negative CSF testing, which limits the ability to definitively classify this as VGCC-antibody-mediated encephalitis. The absence of antibody subtype specification (P/Q-type vs N-type) further restricts precise clinicopathological correlation. Although normal cerebrospinal fluid parameters, neuroimaging, and EEG findings do not exclude autoimmune encephalitis, they have been reported in a subset of cases. Additionally, radioimmunoassay and tissue-based indirect immunofluorescence testing were not performed, which may have improved antibody detection sensitivity. Despite these limitations, the temporal relationship between symptom progression and partial immunotherapy response, along with the relapsing-remitting course, supports a possible immune-mediated mechanism. In accordance with the criteria proposed by Graus et al. (2016), a diagnosis of possible autoimmune encephalitis may be established in patients with a subacute onset of prominent psychiatric or cognitive symptoms, provided that alternative diagnoses have been reasonably excluded, even in the presence of normal MRI, EEG, and cerebrospinal fluid findings. The favorable clinical response to immunomodulatory therapy further supports the diagnosis of VGCC-antibody-associated autoimmune encephalitis.

## Conclusions

This case illustrates a rare and atypical presentation of VGCC antibody-associated autoimmune encephalitis occurring without evidence of an underlying malignancy and manifesting predominantly with psychiatric symptoms in the absence of overt neurological deficits. Such non-classical presentations present substantial diagnostic challenges and carry a high risk of misdiagnosis as primary psychiatric disorders. Importantly, the absence of classical neurological features should not exclude autoimmune encephalitis from consideration.

Early recognition requires a heightened index of suspicion, particularly in patients with acute or subacute onset of atypical, progressive, or treatment-resistant psychiatric manifestations. In this patient, detection of VGCC antibodies was central to establishing the diagnosis and guiding immunotherapy, resulting in significant clinical improvement. This report adds to the limited existing literature on VGCC antibody-associated autoimmune encephalitis and contributes to the growing recognition of its expanding neuropsychiatric spectrum, particularly in the Indian clinical context.
